# The Archives of Clinical Surgery

**Published:** 1861-01

**Authors:** 


ART. XI.—*Archiv für Klinishe Chirurgie.* Erster Band, erstes Heft. Mit 4 Tafeln Abbildungen. Berlin, 1860.

THE journal, the title of which is here given, is edited by Dr. Langenbeck, the distinguished Professor of Surgery in the University of Berlin, with the assistance of Dr. Billroth, Professor of Surgery at Zürich, and Dr. Gurlt, Lecturer on Surgery at Berlin. The volume just issued is the first number of the work, being a portly octavo of nearly three hundred pages, illustrated with four beautiful lithographic plates. The gentlemen engaged in conducting it are all well known for their scientific attainment and large experience, and we have therefore every assurance that it will not have a mere ephemeral existence, but that it is destined to be long-lived, and to take at once a high rank among the leading periodicals of the day. So far as we know, it is the only journal in the world devoted exclusively to clinical surgery.

The principal portion of the volume is occupied with essays on Injuries of the Veins and Tumors of the Sheaths of the Vessels, by Dr. Langenbeck; on Fibroid and Sarcomatous Tumors, by Dr. Sanftleben; and on Resection in the Hip-joint, by Dr. Fock. In addition to these, there are interesting papers on the operation for hare-lip, osteotomy of the tubular bones, ozæna cured by creosote, deformity of the foot, and urinary calculi external to the urinary passages.

The "Archives of Clinical Surgery" will, we trust, meet with an extensive circulation in this country, where the German language is now so much studied by physicians. The work is issued quarterly, and is printed upon excellent paper, with a remarkably clear Roman type; the whole presenting a highly creditable appearance. We wish our new contemporary a long and prosperous career.

